# Miniplate-Aided Mandibular Dentition Distalization as a Camouflage Treatment of a Class III Malocclusion in an Adult

**DOI:** 10.1155/2018/3542792

**Published:** 2018-03-12

**Authors:** Zaki Hakami, Po Jung Chen, Ahmad Ahmida, Nandakumar Janakiraman, Flavio Uribe

**Affiliations:** ^1^Department of Preventive Dental Sciences, Division of Orthodontics, College of Dentistry, Jazan University, Jazan, Saudi Arabia; ^2^Division of Orthodontics, School of Dental Medicine, University of Connecticut, Farmington, CT, USA; ^3^Georgia School of Orthodontics, Atlanta, GA, USA; ^4^Division of Orthodontics, Department of Craniofacial Sciences, School of Dental Medicine, University of Connecticut, Farmington, CT, USA

## Abstract

This case report describes orthodontic camouflage treatment for a 32-year-old African American male patient with Class III malocclusion. The treatment included nonextraction, nonsurgical orthodontic camouflage by en masse distalization of the mandibular teeth using skeletal anchorage devices. The total treatment time was 23 months. Normal overjet and overbite with Class I occlusion were obtained despite the compensated dentition to the skeletal malocclusion. His smile esthetics was significantly improved at the completion of his treatment.

## 1. Introduction

A skeletal Class III malocclusion is an uncommon, yet challenging, orthodontic problem accounting for 8% to 22% of all orthodontic patients [1]. The management of skeletal Class III problems in the late adolescent and adult dentition often involves orthognathic surgery or orthodontic camouflage treatment, which can include differential extraction patterns depending on the severity of the skeletal discrepancy and the patient's expectations and cooperation [2]. Nevertheless, it becomes more challenging when a patient refuses any surgical intervention or extraction treatment options.

Mandibular arch distalization is a nonextraction camouflage treatment modality for Class III malocclusion, and the introduction of skeletal anchorage devices has enabled its use with minimal patient compliance and reciprocal side effects [3–5]. Inter-radicular miniscrews are the most commonly used forms of skeletal anchorage; however, they are often problematic in the mandible because of their high failure rate in the posterior region [6]. Also, the location of miniscrews between the roots limits the extent of distalization unless relocated periodically [7]. In order to avoid these issues, some clinicians place miniscrews extraradicularly in the buccal shelf area or in the retromolar area [8, 9]. On the other hand, some clinicians prefer mandibular distalization by using indirect anchorage with Class III elastics extending to a miniscrew placed in the posterior region of the maxilla [3, 10].

Miniplates are very stable skeletal anchorage devices as they are supported by two or more miniscrews [11]. Sugawara et al. have placed these behind the mandibular second molars, which allowed sufficient mandibular distalization while using high forces in adult patients [5]. Nevertheless, there is a scarcity of case reports in the literature using this approach, particularly in patients with severe Class III malocclusion and financial concerns.

This case report presents an orthodontic camouflage treatment of an adult patient with a pronounced skeletal Class III malocclusion who did not accept surgical or extraction treatment options. Miniplates were used to retract the lower teeth to achieve acceptable dental occlusion with normal overjet and overbite, and favorable lower lip changes.

## 2. Diagnosis

**Figure 1:**
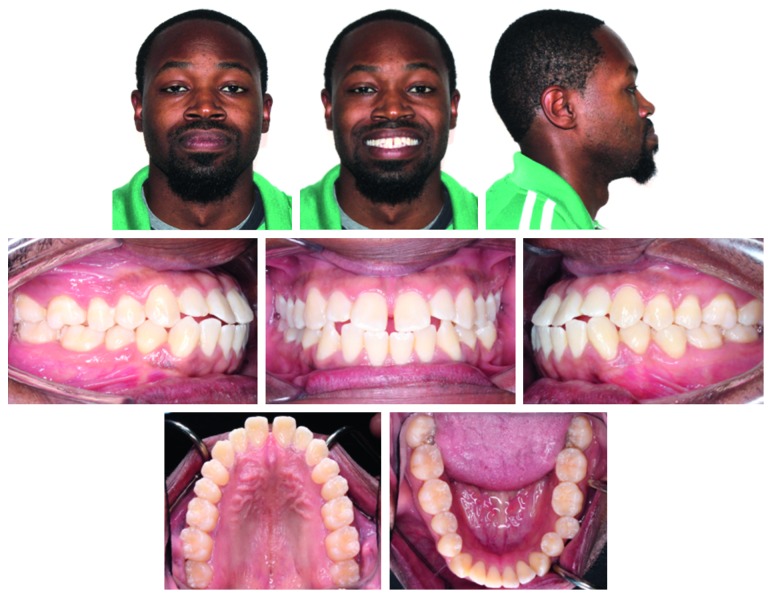
Pretreatment facial and intraoral photographs.

A 32-year-old African American male presented to the university clinic with a chief complaint of dissatisfaction with his dental alignment. The patient's medical history was positive for a latex allergy, but there were no other contraindications for orthodontic therapy. The extraoral examination (Figure 1) showed that he had a dolichofacial, symmetrical face and a prognathic mandible with a Class III appearance. The profile showed reduced facial convexity with anterior divergent face, protrusive upper and lower lips, and an acute nasolabial angle (Figure 1). Temporomandibular joint evaluation exhibited normal signs and symptoms, and midline was coincident with the facial midline.

**Figure 2:**
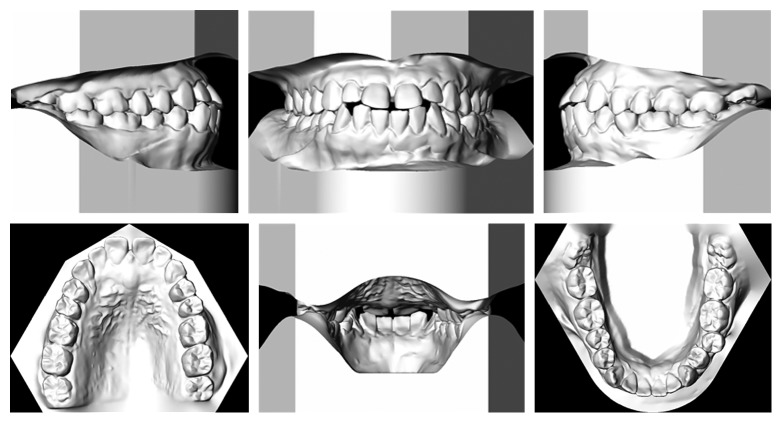
Pretreatment dental casts.

**Figure 3:**
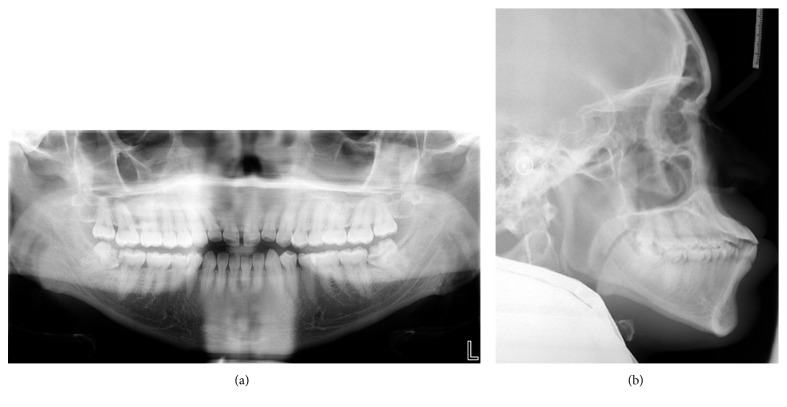
Pretreatment radiographs: (a) panoramic radiograph; (b) lateral cephalograph.

The intraoral examination (Figures 1 and 2) showed a Class III molar relationship (half cusp) and Class III canine tendency bilaterally. Other significant findings included an anterior edge-to-edge relationship and dental crossbite on the lower left second premolar. The dental casts (Figure 2) showed 2 mm maxillary midline diastema and mild crowding (3-4 mm) in the mandible. The panoramic radiograph did not reveal any significant pathology or dental caries, except for a small radiolucent lesion on the distal aspect of the lower right second molar. His mandibular third molars were impacted horizontally (Figure 3(a)). The lateral cephalometric analysis (Figure 3(b) and Table 1) indicated a skeletal Class III jaw relationship (Wits appraisal of −3.5 mm) with bialveolar dental protrusion and increased lower anterior facial height. The maxillary and mandibular incisors were proclined resulting in decreased interincisal angle. In general, the patient was diagnosed with a skeletal Class III malocclusion with bialveolar dental protrusion.

## 3. Treatment Objectives

Based on the problem lists and the patient's concerns, the treatment objectives were to (1) distalize both arches to improve the protrusive lips profile, (2) achieve Class I molar and canine relationships, (3) establish a normal interincisal relationship, and (4) align the teeth and close upper diastema.

## 4. Treatment Alternatives

Several treatment options were considered and presented to the patient. The first alternative was combined orthognathic surgical and orthodontic treatment. Maxillary first premolars would be extracted for anterior retraction, and the anterior crossbite would be corrected with a mandibular setback. This approach would have corrected the skeletal discrepancy and improved the facial and dental esthetics. However, the patient refused this surgical plan due to financial reasons and potential surgical complications.

The second alternative was orthodontic treatment with extraction of four premolars and skeletal anchorage. In this plan, the incisor angulation would be corrected and lip protrusion improved through the retraction of the anterior teeth. However, the patient declined extraction of teeth.

Therefore, the patient's treatment plan would entail nonextraction camouflage treatment by mandibular distalization and skeletal anchorage in both arches or Class III elastics with interproximal reduction on the lower anterior teeth. After discussing these options with the patient, camouflage treatment with skeletal anchorage for distalization on both arches was adopted. Therefore, the treatment plan involved correcting the incisal relationships, reducing anterior proclination, and improving the lip protrusion by distalization with skeletal anchorage.

## 5. Treatment Progress

Prior to initiating the orthodontic treatment, the patient was referred to his general dentist for extraction of third molars and assessment of caries on the lower second molars. Preadjusted appliance with 0.022 × 0.028-inch slots were bonded and banded on both arches for leveling and alignment. Both arches were leveled with continuous archwires, starting with 0.016-inch nickel-titanium and working up to 0.019 × 0.025-inch stainless steel in 9 months. The lower left second premolar crossbite was corrected with X-elastics during the leveling stage. The upper midline space was closed spontaneously during leveling and alignment.

**Figure 4:**
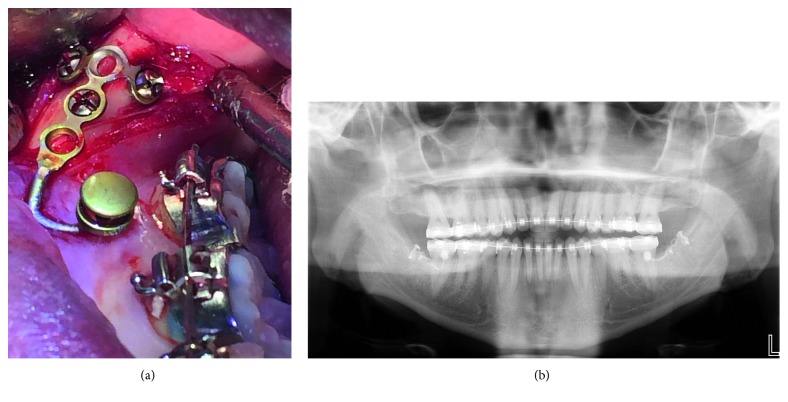
Surgical procedure: (a) intraoral photograph of reflected flap and fixed miniplate; (b) panoramic radiograph after placement of the miniplate.

**Figure 5:**
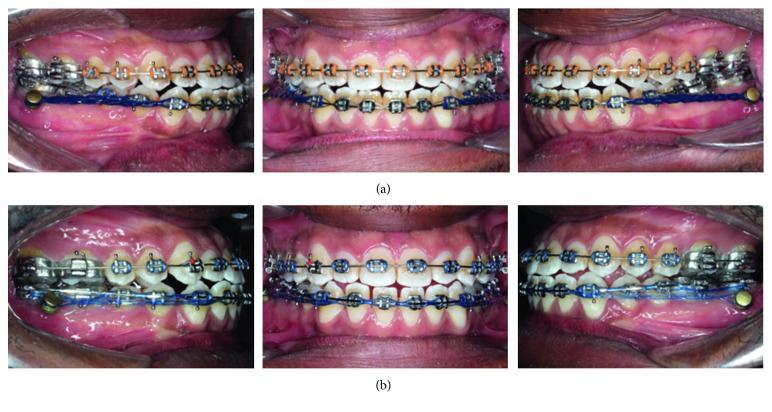
Progress intraoral photographs: (a) miniplates are placed on both sides and en masse distalization has just started; (b) the molars are overcorrected to end-on Class II molar relationship.

The patient was referred to an oral surgeon for miniplate placement after the leveling stage. T-plates (Stryker, Kalamazoo, MI, USA) were placed on the external oblique ridge lateral to the third molar area on both sides and fixed by three miniscrews (1.7 mm in diameter and 5 mm in length). The heads of the miniplates were adjusted to the position between the first and second molars (Figure 4). Two weeks after placement of miniplates, two elastomeric chains exerting 250 gm each were applied from the canine and first premolar to the miniplate on both sides to distalize the mandibular arch on a 0.019 × 0.025-inch stainless steel archwire (Figure 5(a)). The distalization was discontinued after 5.5 months when the molars were overcorrected to end-on Class II molar relationship (Figure 5(b)). Although the molar relationship was overcorrected, the incisal relationship was still edge to edge due to some spacing in the anterior region. A lateral cephalometric film was taken for evaluation, and thin symphysis was noted with the lower incisal roots devoid of labial cortical bone. The patient's mandibular incisors were not suitable for more distal movements because of the thin trabecular bone in the mandibular anterior area that could damage the periodontal tissue. After discussing with patient, the treatment plan was modified to involve only distalization of the mandibular arch instead of both arches.

Thereafter, lower incisor MBT prescription brackets (Opal Orthodontics, USA) were inverted to build up a lingual root torque, and power chain was placed to close the residual anterior spaces. A 0.019 × 0.025-inch stainless steel archwire in the lower arch and a 0.021 × 0.025-inch TMA archwire in the upper arch were placed for finishing. Due to a Bolton discrepancy, a small amount of interproximal enamel reduction was performed on the lower anterior teeth. The total treatment duration was 23 months. The fixed appliances were removed, and retention was provided by maxillary and mandibular lingual-bonded fixed retainers.

## 6. Treatment Results

**Figure 6:**
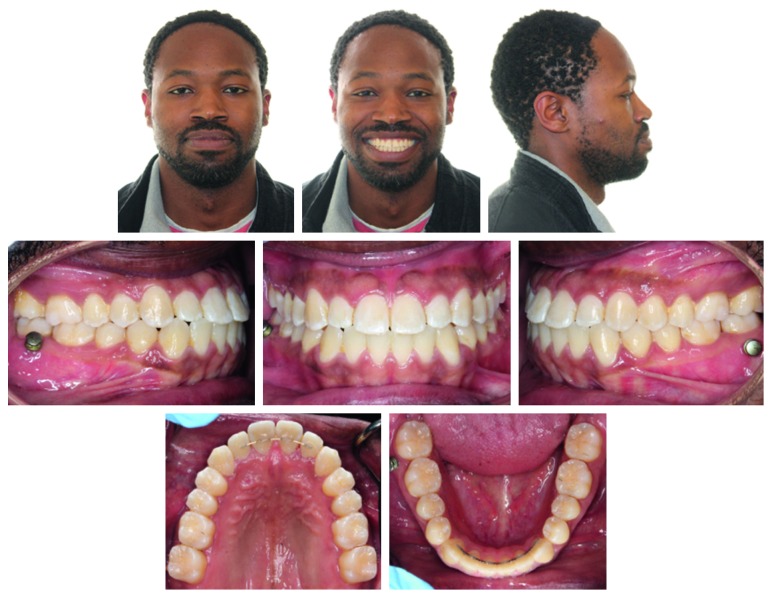
Posttreatment facial and intraoral photographs.

**Figure 7:**
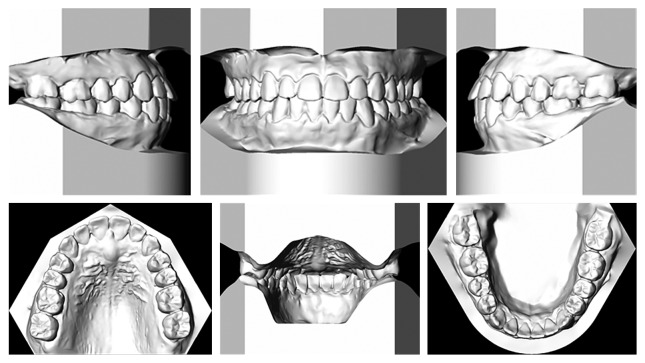
Posttreatment dental casts.

The patient was pleased with the treatment result (Figure 6). The posttreatment records showed improvement of the lower third of the facial profile and retraction of the lower lip with a favorable deepening of the labiomental fold. A Class I canine and molar relationship and normal overjet and overbite were achieved as well as closure of the maxillary midline diastema (Figures 6 and 7).

The transverse dimension was well maintained throughout the treatment. In the maxillary arch, the intercanine width was maintained at 38 mm, whereas the intermolar width was slightly increased from 45 to 45.5 mm. In the mandibular arch, the intercanine width was maintained at 30 mm, and the intermolar width was expanded from 46 to 47 mm (Figures 6 and 7).

**Figure 8:**
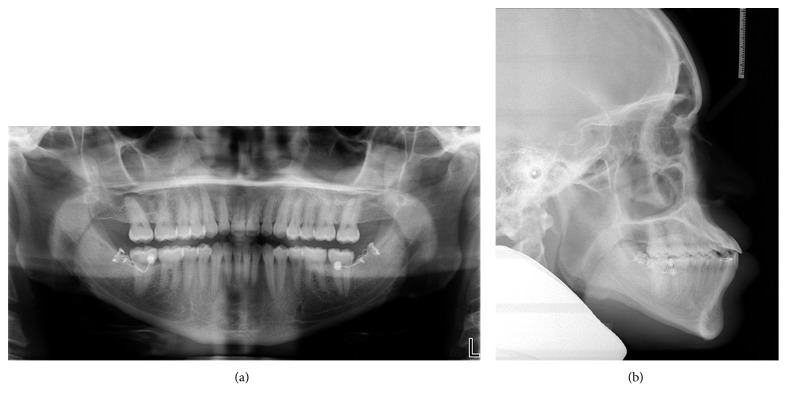
Posttreatment radiographs: (a) panoramic radiograph; (b) lateral cephalograph.

**Figure 9:**
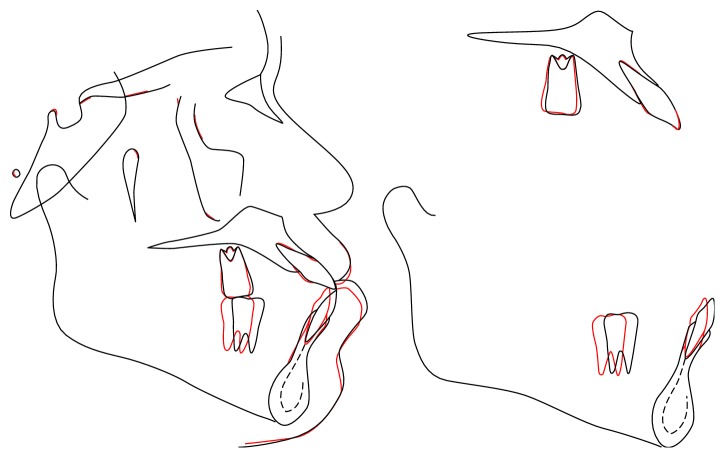
Superimpositions of pretreatment (black line) and posttreatment (red line) cephalometrc tracings.

The posttreatment panoramic radiograph showed good root parallelism with no significant root resorption (Figure 8(a)). Posttreatment lateral cephalometric analysis and superimposition showed that the ANB angle remained unchanged and the interincisal angle increased as the mandibular incisors uprighted. The patient's facial profile, especially the position of the lower lip, was improved. According to the superimposition, the mandibular anterior teeth were retracted about 4 mm with controlled tipping and the mandibular first molars were distalized 4 mm with bodily movement. The maxillary incisors and molars were slightly uprighted (Figures 8(a) and 9 and Table 1).

## 7. Discussion

In this patient, very favorable occlusal and esthetic results were achieved despite the large deviation from the norm in some cephalometric numbers. Ideal overjet, overbite, and Class I relationships were achieved. The dentoalveolar compensatory changes contributing most to the correction of his initial dental and skeletal discrepancies were distal en masse movement of the mandibular dentition with counterclockwise rotation of the occlusal plane.

It has always been debatable whether to treat an adult with a skeletal Class III malocclusion by orthognathic surgery or orthodontic camouflage treatment. As Class III patients show complex interactions of skeletal and dentoalveolar components, statistical techniques, including discriminant analysis, have provided differential diagnoses and treatment options for Class III malocclusion patients by allotting them to a treatment modality more objectively [12–15]. In the studies of Stellzig-Eisenhauer et al., the Wits appraisal was described as the best parameter to discriminate between the two groups [15]. According to their developed formula, this patient would have been treated by combined orthodontic-orthognathic therapy. On the other hand, Benyahia et al. proposed using the Holdaway (H) angle—an angle formed by soft-tissue Nasion and soft-tissue Pogonion—tangent to the upper lip, as the decisive parameter between the two treatment groups [14]. According to this philosophy, this patient would have been successfully treated with orthodontics alone. Although ethnic heterogeneity of the sample has been reported in the latter study [14], a possible explanation of this difference could be that the former study was conducted on white Caucasian subjects [15]. Accordingly, their resulting data might not be directly applicable to African American subjects or other ethnicities.

In general, tooth movement should be maintained within the boundaries of cortical bone. Since the mandible is a horseshoe-shaped bone, distalization of the mandibular teeth is limited anteriorly by the symphysis and posteriorly by the anterior border of the ramus. Kim et al. suggested that the posterior anatomic limit is the lingual cortex of the mandibular body and not the anterior border of the ramus. Furthermore, they indicated that a root of a mandibular second molar is likely to be contacting the inner lingual cortex of the mandible when the posterior available space in lateral cephalograms is lower than 3.9 mm [16]. This patient had greater than 3.9 mm of posterior space after extraction of his impacted third molars, so significant molar distalization could be performed safely. However, the symphysis of this patient was narrow, thus limiting the amount of incisor retraction because of the risks of dehiscence and loss of bone support [17]. Therefore, we changed the initial treatment plan of en masse distalization in both arches to reduce the bimaxillay proclination to only distalization of the mandibular arch while maintaining the maxillary incisor proclination.

Different amounts of mandibular dentition distalization have been reported using various forms of skeletal anchorage. In general, less than 3.5 mm of mandibular arch distalization using miniscrews placed in different sites has been reported [4, 9]. Ye et al. reported more tipping movement of lower molars retracted by indirect usage of miniscrews in the posterior area of the maxillary compared to direct usage of miniscrews in the retromolar area [9]. Using a miniplate, Sugawara et al. reported 3.5 mm distalization of the first molar at the crown level and 1.8 mm at the root level, with a tipping ratio of 46.3% [5]. Recently, Yu et al. showed that the amount of distalization using a ramal plate, a miniplate inserted in the ramus, was 3.2 mm at the crown level and 2.0 mm at the root level, but with a tipping ratio of 37.5% [18]. Miniplates are relatively invasive, requiring flap elevation and suturing in the placement and removal procedures. Thus, miniscrews are widely accepted by both orthodontists and patients [19].

**Figure 10:**
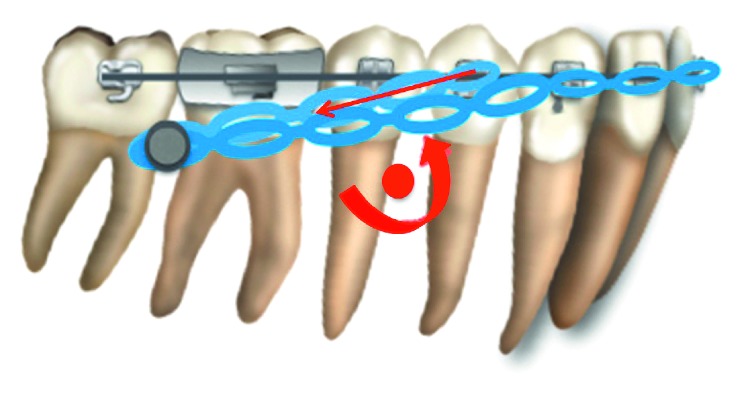
Schematic illustration of the en masse mandibular distalization mechanics. Distalization force applied to the miniplate is above the center of the mandibular dentition. This leads to counterclockwise rotation of the mandibular teeth which helps in correcting the anterior open bite.

In our patient, mandibular molars were distalized by 4 mm with near bodily movement. It is possible that this bodily movement is attributable to the force applied near the center of resistance of the lower arch in combination with a large and stiff working archwire that adequately filled the bracket slot. However, as illustrated in Figure 10, the force vector was still above the center of resistance of the mandibular dentition, leading to a slight counterclockwise rotation of the mandibular arch and an alleviation of the negative overbite as documented previously in similar case reports [20]. Moreover, these results are in agreement with a finite element study by Roberts et al., which demonstrated rotation of the mandibular arch resulted in molar intrusion to reduce the vertical dimension of the occlusion and to close the mandibular plane angle in treating Class III patients with anterior open bite [10]. The cephalogram also showed decreased protrusion and 4 mm retraction of the mandibular incisors with controlled tipping, which improved the labiomental fold and retracted the lower lip.

Despite the fact that this patient declined premolar extraction camouflage treatment, various extraction patterns are possible for orthodontic camouflage of Class III malocclusion in adults. Mandibular incisor extraction is most frequently indicated in mild or moderate Class III malocclusions with an edge-to-edge occlusion of the incisors or anterior crossbite and minimal overbite or open bite [21]. Careful diagnosis and thorough treatment planning should be undertaken to consider the amount of overjet, overbite, anterior crowding, and possible Bolton discrepancies [22]. In this patient, lower incisor extraction could have improved the anterior occlusion but also would have compromised the posterior occlusion and buccal segment interdigitation. Alternatively, bilateral mandibular premolar extraction could have been performed. However, a drawback of this option would be that the maxillary second molars would not have an occlusal contact with the opposing dentition after extraction of the impacted third molars.

With regard to the aforementioned contemplations, intermaxillary Class III elastics would also have been a viable camouflage treatment approach. Yet, undesirable side effects including proclination of the maxillary incisors, extrusion of the molars, and unexpected rotation of the mandible are often associated with intermaxillary Class III elastics [23]. This approach would not have been beneficial for our patient because it would have resulted in exacerbating the proclined maxillary incisors. It has also been suggested that excessive use of Class III elastics might be an etiologic factor on temporomandibular disorders because it might exert upward and backward pressures on the mandible [24]. In this case, Class III elastics were not used during the entire treatment period. Therefore, with minimal pressure on the condyle, en masse distalization of the mandibular dentition using miniplates was a quite effective camouflage treatment approach without any further excessive proclination of the maxillary incisors.

Posttreatment stability of orthodontic treatment should always be taken into consideration during treatment planning of Class III patients. It has been suggested that relapse is positively correlated with the amount of tipping or tooth movement in any direction [5, 18]. Sugawara et al. reported 0.3 mm of relapse one year posttreatment for 3.5 mm of distalization. Moreover, they found no significant correlation between the amount of relapse and the tipping ratio and the amount of tooth movement [5]. On the other side, Chung et al. reported a significant relapse after 8 months of retention in a case report of a Class III patient treated with distalization of the mandibular dentition due to severe distal tipping of the mandibular molar [25]. In our patient, mandibular molars were distalized with a translatory type of movement. The mandibular molars were overcorrected to end-on Class II molar relationship and then allowed to relapse gradually over a year during the treatment. Also, the dimensions of both arches were maintained, which is also an important factor in posttreatment stability [5].

## 8. Conclusion

This case study demonstrates that in mild-to-moderate Class III cases, skeletal anchorage using miniplates is effective in retraction of the whole mandibular dentition leading to correcting the Class III molar relationship and the anterior crossbite without surgery or extraction of premolars. Moreover, this case provides an example that a favorable treatment result, both occlusally and esthetically, can be obtained regardless of a large deviation from the norm in some of the posttreatment cephalometric numbers.

## Figures and Tables

**Table 1:** Cephalometric measurements.

Measurement	Normal	Pretreatment	Posttreatment
SNA (°)	82.0 ± 3.5	88	88
SNB (°)	80.9 ± 3.4	88	88
ANB (°)	1.6 ± 1.5	0	0
IMPA (°)	95.0 ± 7	100	90
U1-NA (mm)	4.3 ± 2.7	16	15
L1-NB (mm)	4 ± 1.8	16	12
Interincisal angle (°)	123.0 ± 6.0	94	106
Upper lip to E-line (mm)	3.0 ± 2.0	0.5	0.5
Lower lip to E-line (mm)	5.0 ± 2.0	9	7
Wits appraisal	−1.0 ± 1.0	−3.5	−2
Occlusal plane-SN (°)	14.4 ± 2.5	8.5	7
SN-MP (°)	33.0 ± 6.0	31	31
FH-MP (°)	26.2 ± 4.5	25	25
U1-SN (°)	108.6 ± 5.5	130	129
U1-NA (°)	22.8 ± 5.7	45	43.5
L1-NB (°)	25.3 ± 6	41	30.5
LFH (ANS-Me/N-Me) (%)	55	59.3	59
